# Assessment of solid microneedle rollers to enhance transmembrane delivery of doxycycline and inhibition of MMP activity

**DOI:** 10.1080/10717544.2017.1337826

**Published:** 2017-06-15

**Authors:** Abbie Omolu, Maryse Bailly, Richard M. Day

**Affiliations:** a Applied Biomedical Engineering Group, Division of Medicine, University College London, London, UK;; b UCL Institute of Ophthalmology, London, UK

**Keywords:** Microneedle rollers, doxycycline hyclate, Franz diffusion cell, drug permeation, Strat-M^TM^ membrane

## Abstract

Many chronic wounds exhibit high matrix metalloproteinase (MMP) activity that impedes the normal wound healing process. Intradermal delivery (IDD) of sub-antimicrobial concentrations of doxycycline, as an MMP inhibitor, could target early stages of chronic wound development and inhibit further wound progression. To deliver doxycycline intradermally, the skin barrier must be disrupted. Microneedle rollers offer a minimally invasive technique to penetrate the skin by creating multiple microchannels that act as temporary conduits for drugs to diffuse through. In this study, an innovative and facile approach for delivery of doxycycline across Strat-M^TM^ membrane was investigated using microneedle rollers. The quantity and rate of doxycycline diffusing through the micropores directly correlated with increasing microneedle lengths (250, 500 and 750 μm). Treatment of Strat-M^TM^ with microneedle rollers resulted in a reduction in fibroblast-mediated collagen gel contraction and MMP activity compared with untreated Strat-M^TM^. Our results show that treatment of an epidermal mimetic with microneedle rollers provides sufficient permeabilization for doxycycline diffusion and inhibition of MMP activity. We conclude that microneedle rollers are a promising, clinically ready tool suitable for delivery of doxycycline intradermally to treat chronic wounds.

## Introduction

Doxycycline hyclate is a tetracycline compound that is used as a broad-spectrum antibiotic in the treatment of a wide variety of bacterial infections including *Chlamydia trachomatis* and gonorrhea (Sloan & Scheinfeld, 2008; Kogawa & Salgado, 2012). At sub-antimicrobial doses, doxycycline is also a broad-spectrum inhibitor of matrix metalloproteinases (MMPs), endogenous enzymes that play an essential role in normal wound healing (Manning et al., 2003). MMPs are produced and secreted by a number of dermal cells including fibroblasts and are responsible for the digestion of the structural extracellular matrix (ECM) components, gelatin and collagen. High levels of MMP activity have been widely documented in the study of the chronic wound microenvironment (Nwomeh et al., 1999; Trengove et al., 1999; Yager & Nwomeh, 1999; Moor et al., 2009; Widgerow, 2011).

Doxycycline has been experimentally shown to reduce tissue degradation (Bildt et al., 2009; Li et al., 2013) and its activity as an MMP inhibitor makes it attractive as a potential treatment for chronic wounds (Naidoo et al., 2009; Stechmiller et al., 2010). The MMP inhibitory action of doxycycline is independent of its antimicrobial properties and thus unrelated to its inhibitory effect on bacterial protein synthesis. Although the full mechanism has yet to be elucidated, it is believed to inhibit enzymatic MMP activity through the chelation of the zinc molecule and MMP gene expression (Li et al., 2013). Doxycycline’s inhibitory activity extends to TNFα-converting enzyme (TACE), a molecule that causes release of tumor necrosis factor-alpha (TNFα), which is an important inflammatory mediator known to impair wound healing (Stechmiller et al., 2010).

The etiopathogenesis of chronic wounds often begins with MMP-mediated damage occurring below the intact skin (Wysoci et al., 1993; Saarialho-Kere, 1998). Therefore, as a potential preventative treatment for chronic wound formation, doxycycline could be delivered intradermally to susceptible intact skin at the earliest stages of wound development. Later stages of wound development may also benefit from the effects of MMP inhibition but are more likely to require invasive surgical intervention as ultimate treatment.

The primary barrier to intradermal drug delivery (IDD) and transdermal drug delivery (TDD) is the stratum corneum (SC) which forms the outermost layer of the epidermis (Vandervoort & Ludwig, 2008). It is a thin hydrophobic layer of cells, formed mainly of corneocytes, and has a thickness that varies from body site to body site – as thin as nine cell layers on the eyelid and as thick as 50 cell layers on the soles of the feet (Ya-Xian et al., 1999). This barrier makes therapeutic access with IDD to susceptible tissue during the early stages of chronic wounds challenging.

To get larger hydrophilic molecules, such as doxycycline (∼1.03 kDa), across the skin, the stratum corneum barrier must be temporarily disrupted. This can be achieved by using penetration enhancers which chemically or physically alter the skin to transiently increase its permeability (Pathan & Setty, 2009). These include skin pretreatments such as hyaluronidase administration, sonophoresis and iontophoresis (Williams & Barry, 2004; Benson, 2005). An emerging technology indicated in an increasing number of TDD and IDD applications are microneedles, a form of physical enhancer. Microneedles come in a variety of designs and delivery mechanisms depending on the desired application. They can differ in shaft geometry, tip shape and needle material (Wu et al., 2008). These physical parameters govern depth of penetration, rigidity and reusability among other factors. There are several methods by which microneedles can be used to deliver a drug through the skin: the ‘poke and patch’ method using solid microneedles, where a drug-loaded patch is applied onto the micropores (van der Maaden et al., 2012), the ‘poke and flow’ method, a controlled release method of infusion through hollow microneedles (van der Maaden et al., 2012), as well as dissolvable (Kommareddy et al., 2012), biodegradable (Kim et al., 2012) and coated (Vrdoljak et al., 2012) microneedles. For single or smaller dosage amounts such as with vaccines (Kommareddy et al., 2012; Edens et al., 2013) or insulin (Gupta et al., 2009; Jj et al., 2013), a spot application with a microneedle patch may be sufficient for drug delivery. However, to deliver a drug over a larger surface area, such as a region of skin at risk of chronic wound development, a more efficient microneedling technique for permeabilizing skin is required. Microneedle rollers offer a method of achieving barrier disruption over a large surface area but the ability of this approach to facilitate drug delivery relevant to chronic wound treatment has not been explored to date.

Here, for the first time, we demonstrate the feasibility of using microneedle rollers to facilitate diffusion of doxycycline across the skin barrier. To simulate intact skin overlying dermal tissue at risk of developing into a chronic wound, a dermal tissue equivalent consisting of a free-floating fibroblast-populated collagen lattice (FPCL) contraction model was combined with Strat-M^TM^ membrane, a non-animal-derived synthetic membrane, to act as a skin barrier mimetic based on its reported human skin-like permeability properties (Uchida et al., 2015, Chernokalskaya et al., 2014). The permeation of doxycycline was measured quantitatively using a Franz diffusion cell under sink conditions and the biological activity assessed *via* fibroblast-mediated FPCL compared between microneedle-treated and untreated Strat-M™.

## Methods

### Microneedle rollers

**Figure 1.**
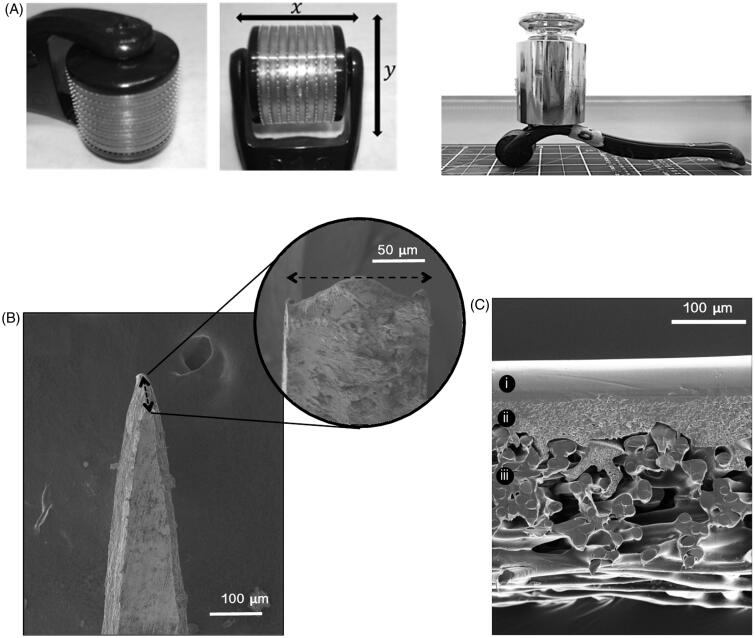
(A) The microneedle roller head, microneedle arrangement and microneedle roller with 500 g mass applied used for static impact insertion of the microneedles with a downward force of 3.8 N. (B) SEM image of a single microneedle tip from a 500 μm microneedle roller. (C) SEM images provide a cross-sectional view of the Strat-M^TM^ membrane, clearly showing its major architectural layers.

Commercially available CE-marked microneedle rollers were supplied in three microneedle lengths, 250, 500 and 750 μm (Sodacoda^TM^, Delmenhorst, Germany). Rollers were composed of two parts, a detachable head containing the microneedles and a handle for holding and manual insertion (Figure 1(A)). Each roller head consisted of 540 titanium microneedles arranged as nine discs with 60 microneedles each.

### Perforation of Strat-M^TM^ by microneedle rollers

Strat-M^TM^ membranes (Merck Millipore, Billerica, MA) were cut into 23 mm circular discs. To perforate the membrane, the roller was placed onto the membrane and manually rolled back and forward five times along a single axis. In a separate experiment to compare perforation by the microneedle rollers in a standardized static model, a 500 g weight (providing a downward force on the microneedles of 3.8 N) was attached to the roller head before application of the roller to the membrane for 30 seconds (Figure 1(A)).

### Quantification of transmembrane diffusion across Strat-M^TM^ membranes

#### Franz diffusion cells

Transmembrane diffusion of doxycycline across Strat-M^TM^ membranes was measured quantitatively using static Franz diffusion cells (Soham Scientific, Ely, UK). Cells were of modular design and consisted of an upper ‘donor’ compartment and a lower ‘receptor’ compartment. Both chambers had a bore size of 12 mm and could each hold up to 3 ml of fluid. The area of permeation afforded by the cells was 113 mm^2^.

Strat-M^TM^ membranes were cut into discs that overlapped with the edges of the Franz cell compartments to prevent leakage and mounted between donor and receptor chambers with sealant. Temperature-equilibrated receptor fluid (0.01 M PBS) (NaCl 8 mg/ml, Na_2_HPO_4_ 1.15 mg/ml, KH_2_PO_4_ 0.2 mg/ml, KCl 0.2 mg/ml; pH 7.4) was sonicated to remove air bubbles and 3 ml added to the receptor compartment along with a Teflon-coated magnetic stirrer (VWR International, Radnor, PA). Cells were placed in a temperature-regulated water bath on top of a water-resistant magnetic stirring plate (Thermo Fisher Scientific, Loughborough, UK) and stirred at 400 rpm to maintain receptor solution homogeneity. The water bath was maintained at 32 °C (human skin surface temperature of limbs).

Conditions for infinite dose permeation were met by using a concentration of doxycycline hyclate at 10% of its solubility limit (50 mg/ml) deliverable to the receptor compartment. Doxycycline hyclate solution (10 mg/ml) was prepared by dissolving 30 mg of doxycycline hyclate powder (Alfa Aesar, Haverhill, MA) in 3 ml of PBS (pH 7.4). The solution was added to the donor compartment. Donor and receptor compartments were covered with Parafilm^TM^ (Sigma-Aldrich, Poole, UK) to prevent water loss through evaporation. Samples of receptor fluid (200 μl) were collected at 10 min intervals during the first hour, then every hour for the first five hours, and then at 24 hours. Samples were transferred to 1 ml Chromacol^TM^ vials (Thermo Fisher Scientific, Loughborough, UK), wrapped in foil to protect the solutions from light and stored at 4 °C until high-performance liquid chromatography (HPLC) analysis was carried out. Temperature-equilibrated PBS was immediately added back into the receptor compartment to replace the sample volume removed and maintain thermodynamic equilibrium.

#### High-performance liquid chromatography (HPLC)

An isocratic method for detecting doxycycline hyclate dissolved in aqueous solutions was conducted on a modular HPLC system (Agilent HPLC 1200 series, Agilent Technologies, Edinburgh, UK) using a 15 cm × 4.6 nm, 5 μm, RP-amide column (Supelco Analytical, Poole, UK). The mobile phase consisted of 75% (v/v) di-iodinated water (with 0.1% [v/v] trifluoroacetic acid) and 25% (v/v) HPLC-grade acetonitrile (Sigma-Aldrich). System parameters were set with a pump flow rate of 1.0 ml/min, sample injection volume of 10 ml, a method stop time of 10 min and detection wavelength of 273 nm. This method produced a chromatogram with a major analyte peak corresponding to doxycycline at 6 min. A calibration curve was created from serial dilutions of doxycycline solution. This was used to calculate doxycycline concentration from solutions of unknown concentration using the area under the chromatogram peak.

### Free-floating fibroblast-populated collagen lattice (FPCL) assay

Human adult dermal fibroblasts were harvested from a 5 mm punch biopsy excised from the presternal dermis of a healthy adult donor undergoing blepharoplasty surgery. The biopsy was mechanically dispersed, outgrown from the explant and established as a primary *in vitro* culture (Li et al., 2015). Monolayer cultures of fibroblasts were grown in T75 cm^2^ vented flasks in ×1 DMEM (Sigma-Aldrich, Poole, UK) supplemented with 10% (v/v) FBS (Gibco, Paisley, UK), 1% (v/v) L-glutamine (Sigma-Aldrich) and 1% (v/v) penicillin–streptomycin–amphotericin B (Sigma-Aldrich). Their environment was maintained at 37 °C and 5% CO_2_ in a fully humidified incubator. Cells were used between passages five and 15 and were grown to ∼80% confluence before passaging.

Cell monolayers were washed with PBS and detached with trypsin–EDTA (Sigma-Aldrich). The cells were pelleted by centrifugation at 1500 rpm for 5 min and resuspended in FBS. Concentrated medium was prepared by adding (% volume) 73.7% ×10 DMEM (Sigma-Aldrich), 7.4% L-glutamine (Sigma-Aldrich) and 18.9% sodium bicarbonate (Gibco). Concentrated medium (160 μl) was added to 1 ml of type I rat tail collagen (First Link UK Ltd., Birmingham, UK) and the solution neutralized dropwise with 80 μl of 1 M sodium hydroxide (Sigma-Aldrich). 100 μl of 1 × 10^5^ cells resuspended in FBS was added to the collagen solution. The cell-enriched collagen gel solution was cast into 10 mm microwells of uncoated glass bottomed 24-well plates (MatTek Corp., Ashland, MA). Gels were polymerized at 37 °C for 15–20 min to form the FPCLs.

Fibroblast-mediated FPCL contraction was monitored using digital photographs of the FPCLs that were taken on days 1, 3, 6 and 7 with a 16 MP camera. The gel area was measured using the ‘elliptical’ measuring tool in the Java-based imaging software ImageJ^®^ (National Institutes of Health [NIH], Bethesda, MD). Contraction was plotted as a percentage of gel area normalized to the original gel area when cast on day 0 (also equivalent to microwell area). Samples of supernatant (75 μl) were collected 1 hour after initial incubation on day 0, and on days 3 and 7, and stored at −20 °C for MMP activity measurement.

### Mounting Strat-M^TM^ membrane into cell crown inserts

To investigate doxycycline permeation, the Strat-M^TM^ membranes were mounted into cell crowns (Scaffdex, Tampere, Finland). The cell crowns were inserted into individual wells of the 24-well MatTek plate so that the basal surface of the membrane was in contact with the medium (750 μl) bathing the collagen gels. Treatment solutions (250 μl) containing doxycycline (416 μM) were pipetted onto the apical surface of the Strat-M^TM^ membrane. A stock solution of doxycycline hyclate solution (10 mg/ml) was prepared by dissolving 30 mg of doxycycline hyclate powder (Alfa Aesar, Haverhill, MA) in 3 ml of PBS (pH 7.4) and adjusted to a final concentration of 416 μM in PBS. Supernatant beneath the mounted Strat-M^TM^ membrane was sampled from around the collagen gel on days 0, 3 and 7 using a fine needle and syringe and stored at −20 °C until MMP activity measurements.

### MMP activity assay

Total MMP activity was determined using the Abcam FRET-based MMP activity assay kit according to manufacturer’s protocol (ab112147; Abcam, Cambridge, UK). Supernatant (25 μl) collected from collagen gel contraction cultures was transferred to 96-well plates and 25 μl 2 mM *p*-aminophenylmercuric acetate solution added. Plates were incubated at 37 °C for 3 hours. MMP red substrate working solution (50 μl) was added to the samples and the plate incubated at room temperature for 1 hour, protected from light. Fluorescence was measured at Ex/Em 540/590 nm using the FLUOstar Optima plate reader (BMG Labtech, Ortenberg, Germany). The fluorescent output readings from the MMP activity assays are relative values that could be affected by signals from background substances and materials. To correct for this effect, the readings for each treatment group were normalized to a background control that contained just the treatment solutions (no FPCLs), for each day.

### Scanning electron microscopy (SEM)

Membranes, micropores and microneedles were imaged by SEM. Strat-M^TM^ membranes were air-dried and coated with gold palladium. Microneedle roller heads were dismantled before imaging. Samples were mounted onto metal blocks and placed under vacuum in SEM (XL-30 FEG SEM; Philips Electron Optics, Eindhoven, The Netherlands). Images were analyzed in ImageJ^®^. Micropore area was calculated using the ‘polygon selections’ tool to outline the perimeter of the pore and measure the internal area. For each microneedle length, six micropore areas were calculated.

### Optical coherence tomography (OCT)

Microneedles inserted into Strat-M^TM^ membrane by applying a mass of 500 g were imaged using the Michelson Diagnostics EX1301 OCT (Michelson Diagnostics, Kent, UK). The 2D images were analyzed in ImageJ^®^.

### Statistical analyses

A two-way ANOVA with Tukey correction for multiple comparisons was carried out to determine statistical significance of percentage gel contraction and MMP activity between treatment groups for each day. Where appropriate, results are expressed as mean ± standard error of the mean (SEM) and in all instances, *p* < .05 denotes a statistically significant difference.

## Results

### Microneedle and Strat-M^TM^ membrane structure

The ultrastructure of the microneedle tips and shafts of the roller systems were visualized using SEM (Figure 1(B)). Strat-M^TM^ membrane thickness, measured by analysis of SEM images, was 300 ± 2.7 μm. Cross-sectional images showed Strat-M^TM^ had two main architectural layers – a smooth upper layer that became increasingly porous and had a layer thickness of 108 ± 2.8 μm, and a thicker highly porous lower layer composed of bundles of thick synthetic fibers measuring 192 ± 5.8 μm (Figure 1(C)).

### Pore formation in Strat-M^TM^ membrane

**Figure 2.**
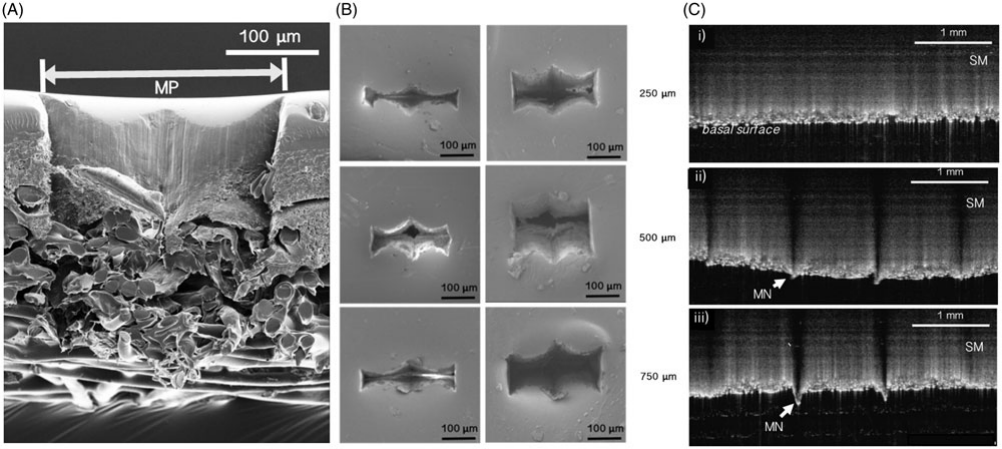
(A) SEM image of a micropore [MP] in Strat-M^TM^ membrane after application of a 500 μm microneedle roller. (B) SEM images of micropores in Strat-M^TM^ membrane after application of a 500 g mass onto microneedle rollers providing a downward force of 3.8 N (left), and micropores in Strat-M^TM^ membrane after unilateral manual rolling with the microneedle rollers (right). (C) OCT images showing the 500 and 750 μm microneedle rollers [MN] penetrating the full thickness of the Strat-M^TM^ membrane [SM]. The 250 μm microneedles did not penetrate through the basal surface of the membrane. Artifacts are caused by the light casting shadows beyond the microneedle tips.

Maximum contact area with the membrane was nine microneedles per row along the x-axis, four microneedles per row along the y-axis when force was applied perpendicularly and infinite microneedles along the y-axis when rolled. SEM allowed the morphology of the micropores created in the Strat-M^TM^ to be examined in greater detail (Figure 2(A)). Due to the tip shape of the microneedles, the pores had an atypical modified diamond shape. Average micropore areas were 11,200 ± 1400, 14,600 ± 2700 and 17,100 ± 3000 μm^2^ (static impact insertion), and 26,800 ± 1400, 29,600 ± 1500 and 36,100 ± 2700 μm^2^ (manual rolling) for 250, 500 and 750 ?m microneedle lengths, respectively. Although pore size increased with increasing microneedle length, a statistically significant difference in micropore area was only observed between the 250 and 750 μm microneedles for static impact insertion (*p* < .001) (Figure 2(B)).

Depth penetration of the roller tips was evaluated using OCT to assess which skin layer the microneedles would reach. The tomographic images show that the 500 and 750 μm length microneedles completely penetrated the 300 μm thick Strat-M^TM^ (Figure 2(C)). The 750 μm microneedles protruded further than the 500 μm microneedles with a 170 μm tip protrusion compared with 80 μm, suggesting that 750 μm microneedles would reach deeper in full-thickness skin. The 250 μm microneedles were shown to enter the Strat-M^TM^ but to not fully penetrate the membrane.

### Pharmacokinetic profile of doxycycline delivery through Strat-M^TM^ membrane

**Figure 3.**
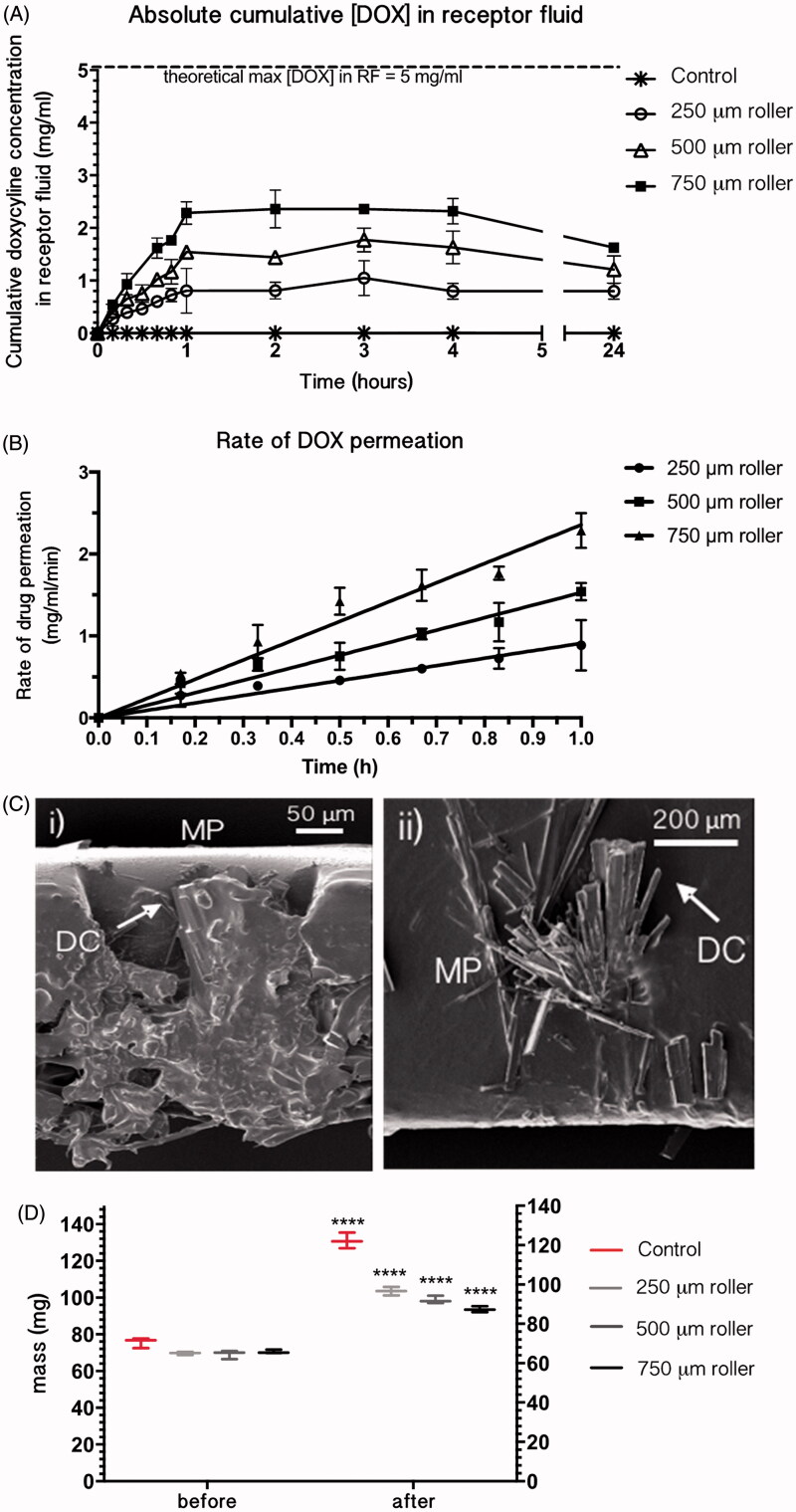
Effect of microneedle application on doxycycline permeation through Strat-M^TM^ membrane (*n* = 4): (A) Cumulative concentration of doxycycline in receptor compartment fluid after treatment with 250, 500 and 750 μm microneedle lengths. (B) Rate of drug permeation from linear regression of cumulative doxycycline concentration during the hour-long lag period. Error bars represent the standard error of the mean. (C) SEM images of doxycycline crystals [DC] retained in and on the Strat-M^TM^ membrane shown occluding micropores [MP] in Strat-M^TM^ membrane treated with 500 μm rollers (left) and doxycycline crystal formation on the membrane surface (right). (D) Box and whisker plots comparing the dry mass of membrane before microneedle permeation (‘before’) with the mass after the permeation study and post-desiccation (‘after’). ‘Control’ represents the membrane samples which had not been treated with rollers; **** = *p* < .0001.

The permeation of doxycycline through untreated and microneedle-treated Strat-M^TM^ was investigated. Cumulative doxycycline concentration was monitored in the receptor fluid over 24 hours. No doxycycline was detectable in the receptor fluid of untreated membranes, indicating that Strat-M^TM^ alone inhibited doxycycline permeation (Figure 3(A)). Conversely, doxycycline was detectable in the receptor fluid in membranes treated with all microneedle lengths after 10 min. The lag time (the time taken for the doxycycline to achieve steady state flux through the membrane) was one hour across all microneedle lengths. Differences between the measured cumulative permeated drug concentrations were statistically significant between all microneedle lengths, with 750 μm microneedle-treated membranes having the greatest rate of drug permeation and 250 μm microneedle-treated membranes the least. Total doxycycline permeation at steady state flux was 0.8, 1.6 and 2.2 mg/ml and rate of doxycycline permeation during lag time was 0.88, 1.53 and 2.30 mg/ml/min for 250, 500 and 750 μm microneedle lengths, respectively (Figure 3(B)). Dose depletion of doxycycline was exhibited up to 24 hours.

The total theoretical doxycycline concentration deliverable to the receptor fluid was 5 mg/ml (half of that in the donor fluid), but the maximum concentration reached was 2.2 mg/ml when Strat-M^TM^ was permeabilized with the 750 μm microneedle rollers. This is possibly due to drug retention in and on the membrane as indicated by SEM images and membrane mass measurements. Doxycycline crystals were observed within the micropores as well as on the membrane surface after desiccation (Figure 3(C)). Mass change measurements show that after desiccation there was a statistically significant increase in membrane mass for both microneedle-treated and untreated membranes (Figure 3(D)).

### Effect of doxycycline on FPCL gel contraction and MMP activity

**Figure 4.**
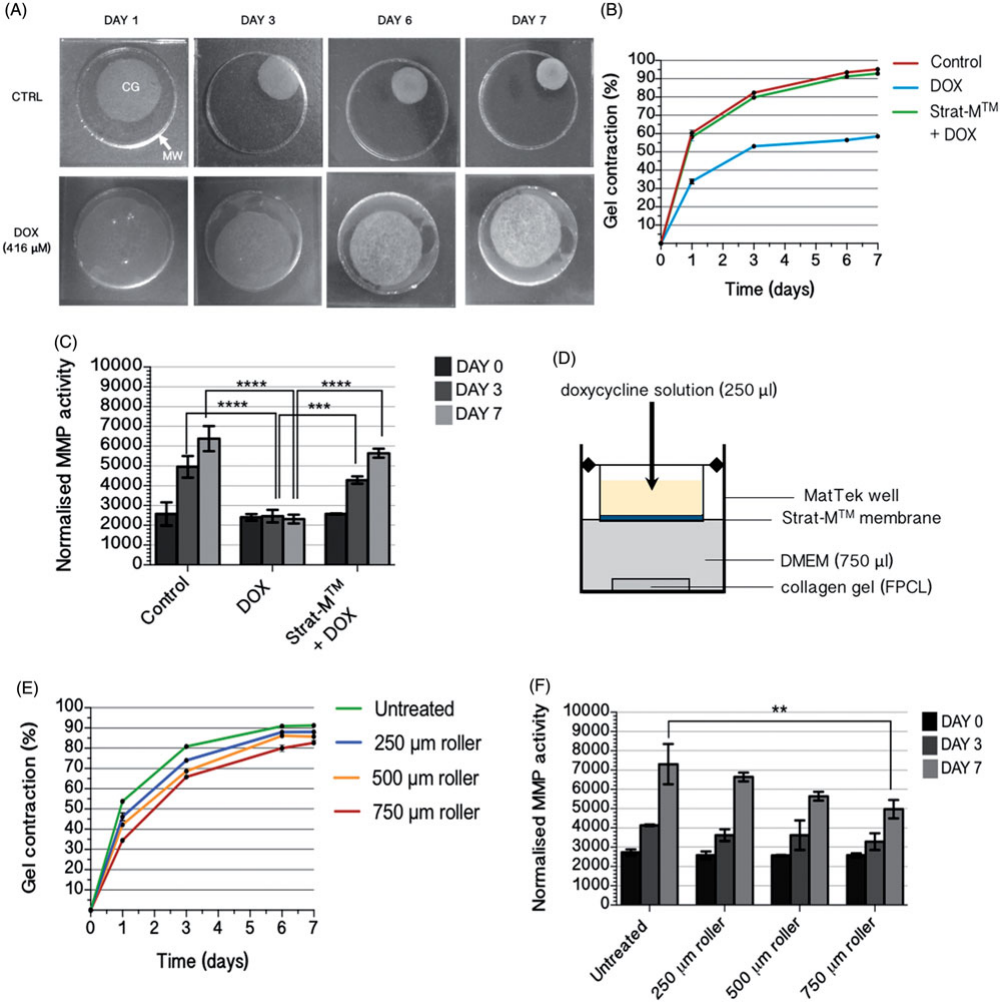
(A) Photographs of gel contraction in FPCLs treated with 416 μM doxycycline and control FPCLs *(n =* 6). The collagen gels [CG] were cast into the plate’s microwells [MW] on day 0 and photographed on days 1, 3, 6 and 7. Percentage gel contraction was calculated by measuring the area of the collagen gel relative to the microwell. Control FPCLs, cultured in complete DMEM only, showed rapid contraction between days 1 and 3. Treatment of gels with 416 μM doxycycline significantly reduced total percentage contraction by day 7 (*p* < .0001). (B) Percentage gel contraction was compared between control FPCLs (‘Control’), FPCLs compartmentalized from 416 μM doxycycline by the Strat-M^TM^ membrane (‘Strat-M^TM^ + DOX’) and FPCLs treated directly with 416 μM doxycycline (‘DOX’) *(n* = 6). Direct treatment with doxycycline resulted in significantly reduced percentage gel contraction compared to control on all days. Strat-M^TM^ was able to significantly inhibit the delivery of 416 μM doxycycline to the FPCLs over the seven-day experiment by compartmentalizing the doxycycline. Due to this, no statistically significant difference in gel contraction was observed between the ‘Strat-M^TM^ + DOX’ and ‘DOX’ groups. (C) Total MMP activity was compared between ‘Control’, ‘Strat-M^TM^ + DOX’ and ‘DOX’ groups (*n* = 6). In the ‘Control’ group, a significant increase in MMP activity was observed by days 3 and 7 compared to day 0. The ‘DOX’ group exhibited significantly inhibited MMP activity compared to ‘Control’ on days 3 and 7 (*p* < .0001). The MMP activity for the ‘Strat-M^TM^ + DOX’ groups showed no statistically significant difference compared to ‘Control’ over the seven-day experiment but was significantly different to ‘DOX’ on days 3 and 7 (*p* < .0001). Error bars represent the standard error of the mean (*** = *p* < .001, **** = *p* < .0001). (D) Schematic representation of Strat-M^TM^ membrane inserted into a cell crown and mounted into an individual well of a 24-well glass-bottomed plate. The basal surface of the membrane was in contact with the DMEM immersing the FPCL. Doxycycline solution was pipetted onto the apical surface of the membrane. (E) Percentage gel contraction was correlated with the length of the microneedles used to permeabilize the Strat-M^TM^ membrane to the 416 μM doxycycline solution (*n* = 6). Permeabilization of the membrane to doxycycline using the microneedles resulted in reduced gel contraction compared to control, significantly so for 250 μm on days 1 and 3, 500 μm on all days and 750 μm on all days assessed of the seven-day experiment. The magnitude of effect was related to needle length; the 750 μm microneedle-treated membrane resulted in the greatest reduction in gel contraction while the 250 μm resulted in the least. (F) Correlation between MMP activity and Strat-M^TM^ treated with increasing microneedle lengths (*n* = 6). A statistically significant difference was observed only between untreated Strat-M^TM^ and 750 μm microneedle-treated Strat-M^TM^ on day 7. Error bars represent the standard error of the mean (**p < .01).

FPCLs cultured in FBS-supplemented DMEM contracted over a seven-day period (Figure 4(A)). FPCLs exhibited a cumulative gel contraction of 60.7 ± 1.6%, 82.4 ± 0.1%, 93.5 ± 0.03% and 95.2 ± 0.1% on days 1, 3, 6 and 7, respectively (Figure 4(B)). The addition of sub-antimicrobial concentrations of doxycycline (416 μM) to the culture medium reduced gel contraction compared with control groups at equivalent time points (*p* < .0001). For this group, gel contraction was 33.8 ± 1.6%, 53.1 ± 0.2%, 56.5 ± 0.4% and 58.5 ± 0.2% on days 1, 3, 6 and 7, respectively (Figure 4(B)). For control FPCLs, the normalized activity of MMPs was shown to sharply increase over the seven-day experiment and effectively doubled between days 0 and 7 (Figure 4(C)). Treatment of the FPCLs with 416 μM doxycycline significantly inhibited MMP activity in the gel supernatant compared with control FPCLs on days 3 and 7, maintaining them at day 0 activity levels (*p* < .0001).

### Effect of Strat-M^TM^ on doxycycline delivery to FPCLs

The addition of the Strat-M^TM^ membrane to the cell crown insert compartmentalized the doxycycline solution from the FPCLs (Figure 4(D)). This prevented the doxycycline from reaching the FPCLs to inhibit gel contraction and MMP activity, unlike FPCLs treated directly with doxycycline. There was no statistically significant difference in gel contraction between control FPCLs and FPCLs compartmentalized with untreated Strat-M^TM^ on any day; gel contraction values were 58.1 ± 2.1%, 79.7 ± 1.0%, 91.2 ± 0.7% and 92.8 ± 0.2% on days 1, 3, 6 and 7, respectively. There was also no statistically significant difference in MMP activity between these two groups. However, there was a statistically significant difference in both gel contraction and MMP activity between FPCLs treated with doxycycline compartmentalized by Strat-M^TM^ and those treated directly with doxycycline on days 1, 3, 6 and 7 (Figure 4(C)).

### Effect of microneedle application on transmembrane doxycycline delivery to FPCLs

To investigate the effect of microneedle treatment on transmembrane permeation of doxycycline to FPCLs, the Strat-M^TM^ membrane was treated with microneedles of 250, 500 or 750 μm in length and doxycycline solution applied to the membrane’s apical surface. Treatment of the Strat-M^TM^ with the 250 μm microneedles resulted in a statistically significant decrease in gel contraction compared with untreated Strat-M^TM^ on days 1 and 3 (*p* < .0001) but not on days 6 and 7 (Figure 4(E)). Increasing microneedle length resulted in significantly reduced gel contraction for 500 μm (*p* < .001) and 750 μm (*p* < .0001) microneedle-treated membranes compared with untreated membranes on days 1, 3, 6 and 7. There was also a significant difference in gel contraction between 250 μm and 750 μm groups on day 7 (*p* < .001). MMP activity was significantly reduced on day 7 for FPCLs compartmentalized with 750 μm microneedle-treated Strat-M^TM^ (*p* < .01) (Figure 4(F)).

## Discussion

Microneedles, typically ranging from 100 μm to 1 mm in length, sit at the interface between hypodermic needles and transdermal patches (Nava-Arzaluz et al., 2012). They are considered an attractive, minimally invasive method of delivering drugs across the tough SC and function to increase skin permeability by forming temporary channels that act as conduits for drug transit. They are able to deliver a defined amount of drug to a target skin layer and since they are able to deliver drugs locally, can avoid distribution to the whole body *via* systemic circulation, decreasing toxicity and reducing side effects (Trommer & Neubert, 2006). Microneedles also have improved patient compliance when compared with hypodermic needles and have been shown to deliver drugs painlessly and blood-free (Kaushik et al., 2001; Bal et al., 2008; Gupta et al., 2011).

To achieve maximum clinical impact, microneedle-based technologies need to be relatively low cost in comparison with existing mechanical enhancer techniques. Microneedle rollers are already commercially available for cosmetic applications but their utility for clinical purposes in chronic wound treatment has not been explored to date. To investigate how microneedle rollers facilitate delivery of doxycycline across intact skin susceptible to chronic wounds, a novel dual approach to *in vitro* modeling was devised. The recently launched Strat-M^TM^ membrane was used to asses both (i) doxycycline permeation using a Franz diffusion cell system to establish fundamental permeation data under infinite dose conditions and (ii) the biological activity of the diffused drug using a three-dimensional cell-enriched collagen matrix that provided an indication of doxycycline-mediated inhibition of MMP activity.

Micropore area and depth of penetration of the rollers into Strat-M^TM^ membrane correlated with microneedle length. Since Strat-M^TM^ was found to be impermeable to doxycycline, micropores created by the microneedles acted as the sole conduits for drug permeation. As a consequence, the longer microneedles resulted in larger micropores and a greater rate of doxycycline diffusion, with higher cumulative concentrations of doxycycline in the receptor fluid. The reasons for this are likely to be two-fold. Firstly, the longer microneedles created micropores with a greater entry area on the membranes apical surface. Secondly, they were also able to penetrate through the entire 300 μm thick Strat-M^TM^ membrane to a greater depth, creating a longer micropore annulus into the target region than shorter microneedles that did not fully penetrate the membrane. Previous studies comparing the effect of different microneedle lengths have also shown a correlation between increasing microneedle length and increased drug permeation (Park et al., 2010; Kim et al., 2012). In this study, it was observed that the maximum length of the 500 μm and 750 μm microneedles did not penetrate the membrane, since there was a residual portion of their shafts that did not make contact with the membrane’s apical surface. Possible explanations for this include neighboring needles preventing maximum depth penetration also referred to as the ‘bed of nails’ effect. Notably, micropore closure and contraction of the skin *in vivo* is a biological behavior that cannot be mimicked by the acellular Strat-M^TM^. The resealing kinetics of micropores created by these microneedle rollers in human skin need to be investigated to give an indication of the timeframe within which doxycycline could be delivered intradermally, without the use of drug delayers, during clinical application.

Rolling application of the microneedles resulted in larger micropores compared with static impact insertion of the microneedles using 3.8 N of perpendicular force. Rolling involves shearing forces that disrupt the membrane along the y-axis that are not associated with perpendicular application of force. The extent of membrane disruption by manual rolling may be dependent on the number of rolls as well as the number of planes in which the microneedles are rolled in. In this study, the microneedles were rolled unilaterally without removal of the microneedles from the membrane; however, other studies have used multilateral rolling which creates overlapping micropores (Badran et al., 2009; You et al., 2010; Zhou et al., 2010). This suggests that for ‘poke and patch’ IDD and TDD, rolling is a more efficient way of permeabilizing skin than static impact insertion of the microneedle system, and that repeated rolls increase drug diffusion rates (Park et al., 2010). However, the force applied with manual rolling in this study, as well as in any proposed clinical application, is likely to have been inconsistent due to intra- and interoperator variation. The impact of this, in terms of consistency of dose delivery, will require further investigation.

Previous studies have shown that doxycycline-mediated inhibition of MMP activity in FPCL models results in reduced gel contraction and MMP activity (Li et al., 2013). Therefore, a similar system was used to verify biological activity of doxycycline after its permeation through microneedle-treated Strat-M^TM^. The concentration of doxycycline used in this study (416 μM) matched the optimal concentration shown to reduce fibroblast-mediated gel contraction of FPCLs (Franco et al., 2006; Li et al., 2013). We firstly confirmed that the effect of doxycycline on gel contraction inhibition was linked to a decrease in MMP activity as previously shown (Li et al., 2013), as total releasable MMP activity in the FPCL culture medium was inhibited by 416 μM doxycycline over a period of seven days. When the Strat-M^TM^ membrane was used to mimic skin barrier function by compartmentalizing the FPCL from the doxycycline treatment, it was able to effectively prevent permeation of doxycycline to the underlying FPCL. These findings corroborate the data from the Franz permeation cell study which showed that no doxycycline was present in the receptor fluid when untreated Strat-M^TM^ was placed into the Franz cell. When the microneedle rollers were applied to the Strat-M^TM^, increasing lengths of the microneedles on the roller directly correlated with the extent of Strat-M^TM^ permeabilization to doxycycline over a seven-day period as assessed through reduced FPCL gel contraction and inhibition of MMP activity.

A quantity of the doxycycline solution was retained as crystals within and on the Strat-M^TM^ membrane. These yellow crystals were visible in hydrated membranes immediately after the permeation study, confirming that their presence was not a result of the drying process. They were also not observed when PBS alone was delivered through the membrane. The reason for the crystallization of doxycycline and its deposition on the membrane is unclear; the donor fluid concentration was a fifth of its solubility limit so, theoretically, the solution was unsaturated. Molecules in solution will crystallize when a nucleation point is reached and it is more energetically favorable to form and deposit crystals than remain dissolved (Markov, 2003). Thus, it is possible that there is an interaction between the Strat-M^TM^ surface and dissolved doxycycline molecules that stimulates this process. The presence of these crystals explains why the theoretical concentration of doxycycline in the receptor fluid was not reached – crystallization of doxycycline inside micropores would limit further drug permeation and also reduce the amount of drug available to diffuse. A solvent, such as methanol, could be used to improve doxycycline solubility in the model. However, this is likely to interfere with the Strat-M^TM^ structure and integrity, confounding its true permeation properties since it is structurally sensitive to such chemicals. In *in vivo* human skin, drug crystallization needs to be assessed and may be overcome through the addition of solubilizing agents to the drug. Moreover, therapeutic concentrations of doxycycline hyclate administered by IDD would in reality be much lower than 10 mg/ml, which was used to satisfy infinite dose conditions.

There is great potential for the clinical application of microneedle rollers in chronic wound management, specifically to deliver the active drug in a timely manner to prevent wound development and to treat existing tissue damage. There are several ways this can be achieved. The microneedle rollers could be used to permeabilize the surface of intact skin overlying a very early stage wound, using their high mobility and ability to cover large surface areas. A doxycycline-infused patch or dressing could then be applied onto the skin to promote wound healing. For early stage open chronic wounds, the microneedle rollers could be used in a circumferential manner, by rolling the device around the perimeter of the wound. This process should itself promote re-epithelization, but also permeabilizes the surrounding skin to the active drug in order to help reduce the spread of MMP-mediated damage into the surrounding tissue (Liebl and Kloth, 2012). For hygiene purposes, best clinical practice would be single-use of the roller system given the nature of the disease that the target patient population has, though disinfection and sterilization would be possible for repeated use on a single individual.

## Conclusion

This study has demonstrated the potential utility of microneedle rollers as a facile and readily translatable technology for IDD of doxycycline across intact skin at risk of chronic wound formation. We have outlined the development of a novel *in vitro* model of the human epidermis and dermis by combining Strat-M^TM^ membrane with the FPCL system. This model is simple, economical and reproducible and provides a good indication of how doxycycline treatment combined with microneedle rollers may translate into *in vivo* experiments using clinically relevant models that mimic chronic wound microenvironments.
